# Comparison of Electrophysical Properties of PZT-Type Ceramics Obtained by Conventional and Mechanochemical Methods

**DOI:** 10.3390/ma12203301

**Published:** 2019-10-11

**Authors:** Dariusz Bochenek, Przemysław Niemiec, Izabela Szafraniak-Wiza, Grzegorz Dercz

**Affiliations:** 1 Faculty of Science and Technology, Institute of Materials Engineering, University of Silesia in Katowice, 75 Pułku Piechoty 1a, 41–500 Chorzów, Poland; przemyslaw.niemiec@us.edu.pl (P.N.); grzegorz.dercz@us.edu.pl (G.D.); 2 Institute of Materials Science and Engineering, Poznań University of Technology, Jana Pawła II 24, 61-138 Poznań, Poland; izabela.szafraniak-wiza@put.poznan.pl

**Keywords:** PZT-type ceramics, doped, mechanochemical activation, piezoceramics

## Abstract

In the paper, the multicomponent PZT-type ceramics with Pb(Zr_0.49_Ti_0.51_)_0.94_Mn_0.015_Sb_0.01_W_0.015_Ni_0.03_O_3_ composition have been obtained by conventional and mechanochemical methods. With conventional ceramic technology, PZT-type ceramics have been synthesized by the method of calcination powder (850 °C/4 h). Instead of this step, the mechanochemical synthesis process for different milling periods (15 h, 25 h, 50 h, 75 h) has been applied for a second batch of samples. To obtain the dense PZT-type ceramic samples, powders have been sintered by free sintering method at conditions of 1150 °C/2 h. Studies have shown that the perovskite structure of the PZT-type material is formed during mechanochemical activation of powders during the technological process at low temperature. The application of the mechanochemical synthesis to obtain the PZT-type materials also allows shortening of the technological process, and the useful electrophysical properties of ceramic samples are not reduced at the same time. The presented results have confirmed that the investigated materials can be used in microelectronic applications, especially as elements of actuators and piezoelectric transducers.

## 1. Introduction

The most common ceramic solid solutions, extensively used in the electronic industry, are perovskite ferroelectric oxides with a general formula ABO_3_, for example, Pb(Zr,Ti)O_3_ (PZT). To improve the piezoelectric properties of those materials, various modifications, such as different doping, have been carefully examined. The PZT materials in a Zr/Ti proportion close to 52/48 (known as morphotropic phase boundary, MPB) are most functional for the application point of view, because they exhibit one of the highest values of piezoelectric parameters, that is, electromechanical coupling factor *k_p_* (0.53–0.65) and piezoelectric coefficient *d_33_* (250–325 pC/N) [1,2,3,4,5,6,7,8]. Other merits of the PZT materials are their moderate permittivity, low dielectric losses, and high piezoelectric factors (*k_p_*, *k_t_*, *k_ij_*) which make them interesting for actuators, piezoelectric transducers, microsensors, ultrasonic transmitters and receivers, sound transducers, microphones, and so forth [9,10,11,12,13,14,15,16]. PZT powders are usually synthesized by the solid-state reaction process (i.e., calcination method) using mixed oxides as starting materials. Also, the conventional solid-state reacted PZT powders are sintered at very high temperatures [17]. It has been shown that the chemical reactivity of starting materials could be improved remarkably after an appropriate milling treatment, for example, high-energy ball milling (HEBM), which is also known as mechanochemical activation or mechanochemical synthesis. Moreover, in many materials, the perovskite phase formation takes place directly during the high-energy milling process [18,19,20,21,22,23,24,25,26]. The fundamental advantage of application of the mechanochemical process during PZT-type ceramic preparation is the avoidance of the multiple steps of technology at upraised temperatures [27,28].

The aim of this work has been the comparison of useful ceramic properties obtained by conventional and mechanochemical methods. The multicomponent Pb(Zr_0.49_Ti_0.51_)O_3_ doped by Mn^4+^, Sb^3+^, W^6+^, Ni^2+^ was chosen, that is, the type of so-called “ferroelectric hard/soft” doping [29] was used (with Sb^3+^, Ni^2+^ “ferroelectric hard” admixtures and W^6+^ “ferroelectric soft” admixture) in order to obtain ceramic materials with optimal piezoelectric properties (average values), but with high time stability of their physical properties. Additionally, Mn^4+^ admixture was aimed at increasing grain homogeneity in the ceramic microstructure.

Both the powder and ceramic preparations were carefully monitored at different preparation stages using differential thermal analysis (DTA), thermogravimetric analysis (TG), and X-ray diffraction (XRD) methods. The final properties of the ceramics samples were examined by scanning electron microscopy (SEM) and DC electrical conductivity tests, testing dielectric, ferroelectric, and piezoelectric properties.

## 2. Experiment

In this work, the multicomponent PZT-type ceramic samples with the chemical compositions Pb(Zr_0.49_Ti_0.51_)_0.94_Mn_0.015_Sb_0.01_W_0.015_Ni_0.03_O_3_ (PZTMSWN) were obtained and investigated. Two fabrication methods were used to obtain ceramic samples: (i) conventional ceramic technology (C) and (ii) mechanochemical synthesis (M). The starting mixture consisted of the following simple oxides: PbO, ZrO_2_, TiO_2_, WO_3_, Sb_2_O_3_, MnO_2_, and NiO. In both cases, a stoichiometric ratio of starting oxides was used. The first stage of the conventional route was the mixing of powders for 24 h in ethyl alcohol using a Fritsch planetary ball mill. Next, the powder mixture was synthesized by calcination method at conditions: *T* = 850 °C for *t* = 4 h. The temperature synthesis was appointed on the basis of thermogravimetric analysis (DTA, DTG, TG) tests using a Q-1500D derivatograph (from 20 °C to 1030 °C). Finally, the sintering (densification) was carried out by the free sintering method at conditions: *T* = 1150 °C for *t* = 2, 3, 4 h (d*T*/d*t* = 150 °C/h). At work, the sample with the best properties (3 h) was chosen to compare the results (marked as C3h). 

In the case of the mechanochemical synthesis, the high-energy ball milling was carried out in the shaker-type 8000 Mixer Mill (SPEX SamplePrep, Metuchen, NJ, USA) at room temperature (in the air atmosphere) [30]. The ball-to-powder weight ratio parameter was kept as 5:1. The oxides were milled for different periods (i.e., 15 h, 25 h, 50 h, and 75 h). The synthesis process was controlled by X-ray diffraction tests at various milling times. The XRD tests were performed on a PANalytical Empyrean X-ray (PANalytical B.V., Almelo, The Netherlands) powder diffractometer (CuK*_α_* radiation, 45 kV, 40 mA). The powders, obtained after mechanochemical synthesis, were used for the ceramic samples’ preparation. The compacting (densification) of the ceramic samples was performed at the same condition as the conventional ones (i.e., by a free sintering method at *T_s_* = 1150 °C for *t_s_* = 2 h). Due to the poor physical quality (not the single phase material) of 15 h milled samples, this series was omitted from further investigations. The ceramics, obtained from mechanochemical synthesised powders, were marked as M25h, M50h, and M75h, respectively. Finally, both batches of the ceramic samples were ground, polished, and annealed at a temperature of 700 °C for 15 min in order to remove mechanical stresses. 

The X-ray diffraction patterns (PANalytical, Phillips X’Pert Pro, Eindhoven, The Netherlands) were registered at room temperature (*T_r_*) in the 2*θ* range: 10° to 80° in steps-scan mode: 0.05° and 4 s/step. The microstructures of the ceramic samples were examined by scanning electron microscope (JSM-7100 TTL LV Field Emission Scanning Electron Microscope, JEOL, Tokyo, Japan). Dielectric properties were measured using a capacity bridge of a QuadTech 1920 Precision LCR Meter (Quad/Tech, Inc., Maynard, MA, USA) in the heating cycle and in the frequency range from 1 kHz to 1 MHz (in the range of 20–450 °C). Hysteresis (*P*-*E*) loops were examined with a Sawyer–Tower circuit and a HEOPS-5B6 precision high-voltage amplifier (Matsusada Inc., Kusatsu, Japan) in the range of 20–110 °C. DC conductivity tests were carried out in temperatures ranging from 20 °C to 440 °C with a 6517B Keithley electrometer (Cleveland, OH, USA).

To calculate piezoelectric constants, the ceramic samples were electrically poled by the high-voltage method using a Matsusada Precision Inc. HEOPS-5B6 high voltage supply (Kusatsu, Japan) (silicon oil, poling field *E_pol_* = 30 kV/cm, poling time *t_pol_* = 0.5 h, at poling temperature *T_pol_* = 120 °C). Piezoelectric parameters were calculated using the resonance–antiresonance method. The piezoelectric coefficient *d_33_* was measured at *T_r_* using a YE2730A d33 meter (APC International Ltd., Mackeyville, PA, USA).

## 3. Results and Discussion

**Figure 1:**
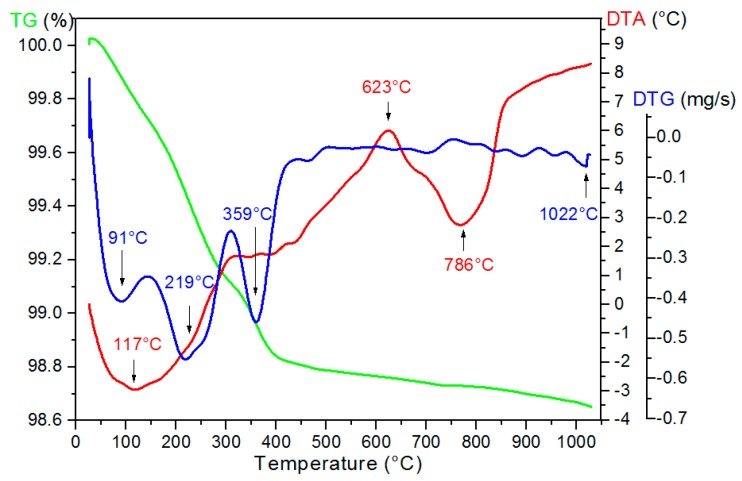
Thermogravimetric analysis of the PZTMSWN powder (C3h sample).

The properties of ceramics are closely related to preparation conditions. In the case of conventional synthesis, the appropriate thermal treatment (especially temperature) is the one of the most important factors. In the case of the volatile reagents (e.g., PbO at high temperature), it is necessary to lower the calcination/sintering temperature to avoid any stoichiometric (and structural) changes. One possible method to determinate the correct preparation condition is based on DTA/TG analyses. The mixture of the starting oxides, used also in conventional ceramic technology, was studied by DTA/TG analyses (Figure 1). On both the TG and DTA curves, several anomalies can be observed. The TG anomalies are related with the mass loss of the sample during the heating cycle. For the investigated PZTMSWN powder, the largest weight loss was observed near 400 °C and the total loss of mass was 0.3% in the measured temperature range from 20 °C to 1030 °C. Further details are clearly visible on the DTG curve. It shows the change in the rate of decomposition of the tested powders with the rise of temperature. The several processes corresponding to local minima on the DTG curve are visible on the graph. The first one, visible as minima below 120 °C on the DTG curves, is associated with loss of water moisture from the sample surfaces. The DTG minima are associated with the changes occurring on the DTA curve. The strong peak, visible at 623 °C on the DTA curve, is related to the formation of undesirable phases (the pyrochlore phase) in the PZT material. Fast temperature increase during the synthesis process prevents the formation of this unnecessary parasitic phase. Further minima are related to creation of the perovskite PZT-like structure and other intermediate phases. The DTA results have shown that the synthesis process of the ceramic materials is realized at 786 °C. Based on this research, the synthesis of the ceramic powders was carried out at *T_synth_* = 850 °C and *t_synt_* = 4 h.

**Figure 2:**
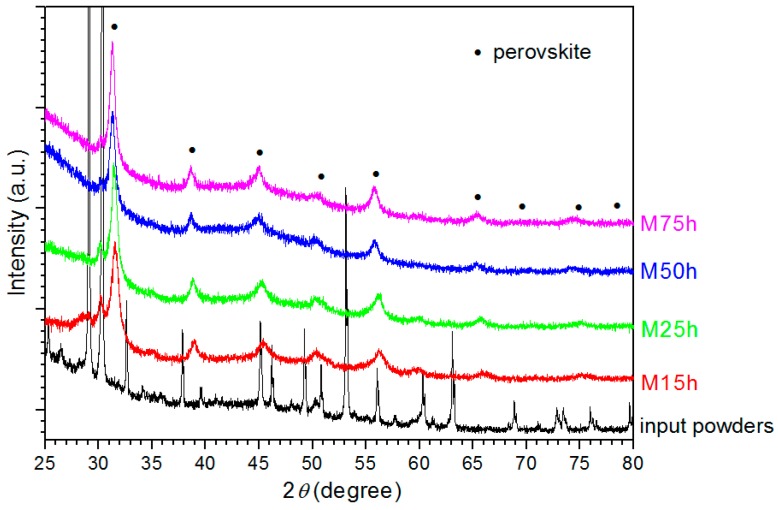
X-ray diffraction patterns of the powders after different milling periods (15 h, 25 h, 50 h, and 75 h). The peaks related to the perovskite are marked by ●.

In regard to the mechanochemical synthesis, the mills are kept at ambient condition, however, local temperature can rise due to the collisions with balls. Among several parameters having impact on the synthesis process, the milling duration is the most important one. To monitor the synthesis development, the powders after different milling durations were examined by XRD measurements (Figure 2). For the investigated powders, the perovskite phase appeared directly during milling (without any additional thermal treatment). The perovskite phase was already present for the powders milled for 15 h. However, the powder included also secondary phases (beside the diffraction peak related to the perovskite phase, there are other peaks present on the diffraction patterns). As a result of the longer milling, the amount of the perovskite phase increased, which is associated with sharpening of characteristic peaks. 

**Figure 3:**
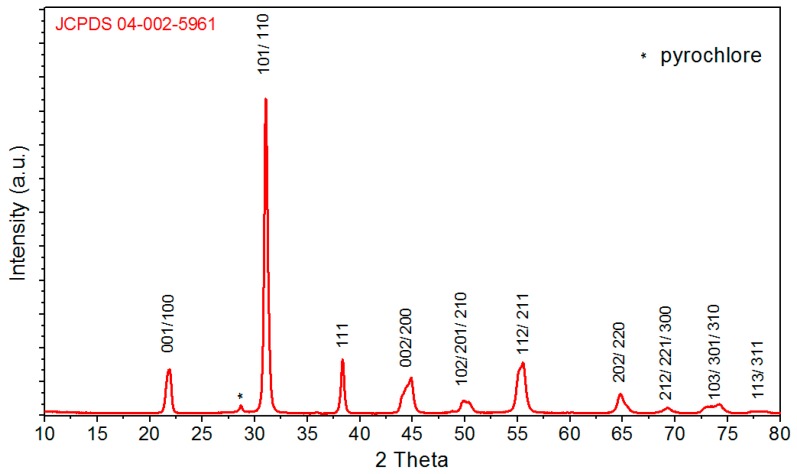
X-ray diffraction patterns of powders of the C3h sample.

The X-ray diffraction pattern of the PZTMSWN material is shown in Figure 3 (at room temperature). The diagram shows strong peaks related to the perovskite phase. The diffraction peaks matched to JCPDS card No. 04-002-5961 pattern with tetragonal perovskite structure and P4mm space group *a_0_* = 4.03, *b_0_* = 4.03, *c_0_* = 4.08. In Figure 3, the graph shows also the trace amounts of the pyrochlore phase.

**Figure 4:**
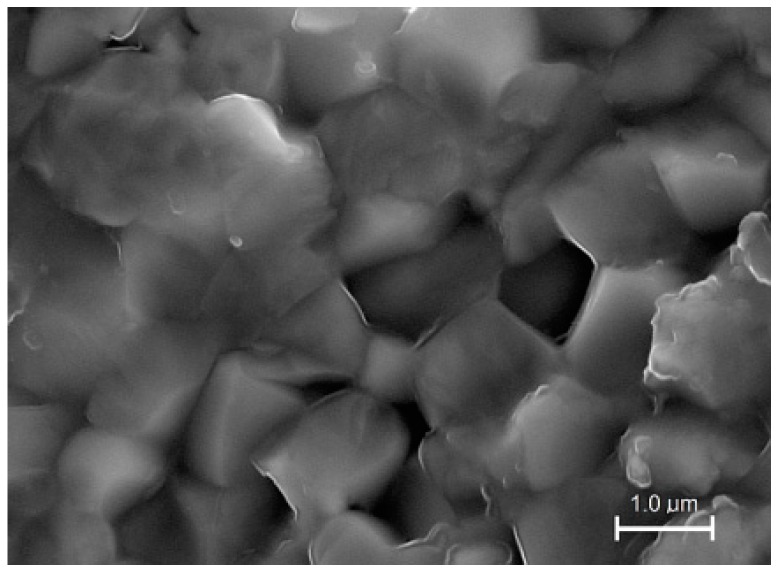
SEM microstructure of the C3h fracture of sample.

**Figure 5:**
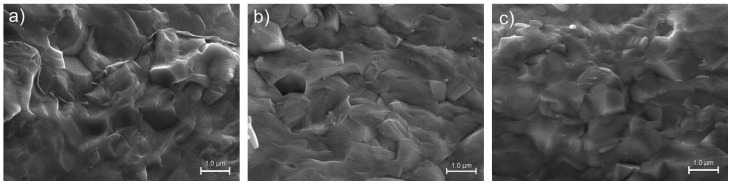
SEM microstructure of the M series ceramic samples (fractures of samples): (**a**) M25h; (**b**) M50h; (**c**) M75h.

The SEM investigations (fractures of samples) were carried out on all samples (Figure 4 and Figure 5). Both batches of the samples had dense structures; however, their morphology was different (shape of grains, grain boundaries, and grain size). In the case of the C3h ceramics, the breakthrough of the sample consisting of grains glued together, without clear grain boundaries. The microstructures of the ceramic samples obtained by mechanochemical synthesis (Figure 5) showed a high degree of sintering and the grains had firmly solidified with each other.

**Figure 6:**
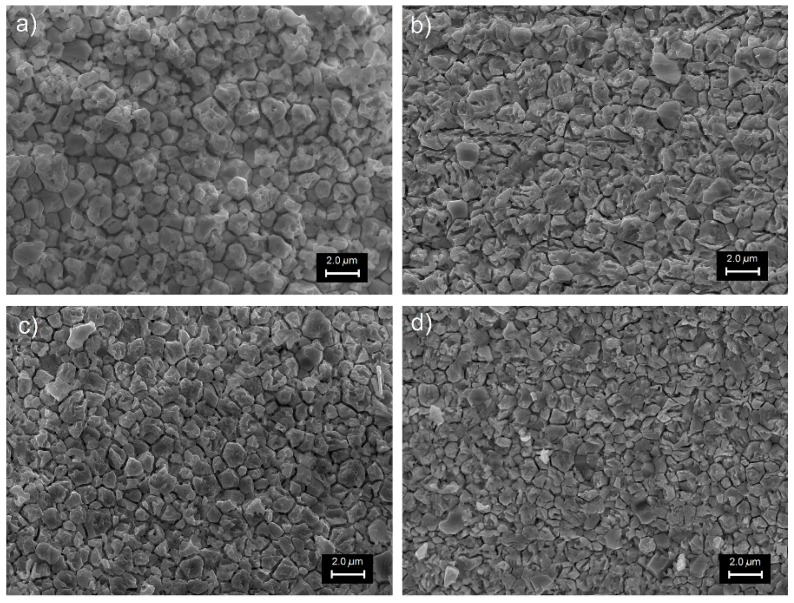
SEM microstructure of the PZTMSWN samples (etched surfaces): (**a**) C3h; (**b**) M25h; (**c**) M50h; (**d**) M75h.

The domain structure of the samples was revealed by fracture surface etching, after they were ground and polished (Figure 6). SEM images show a large heterogeneity of grain shape for a series of samples obtained by mechanochemical activation method (Figure 6b–d) compared to a sample obtained by classical technology (Figure 6a); the largest occurred for the composition with the lowest mixing time in the HEBM method (M25h). It resulted from the specificity and method of mixing powder in the MS method. The average grain size decreased with increasing mechanochemical activation time. For the tested ceramic materials, it was 1.48 μm, 1.52 μm, 1.40 μm, and 1.32 μm for the C3h, M25h, M50h, and M75h samples, respectively.

**Figure 7:**
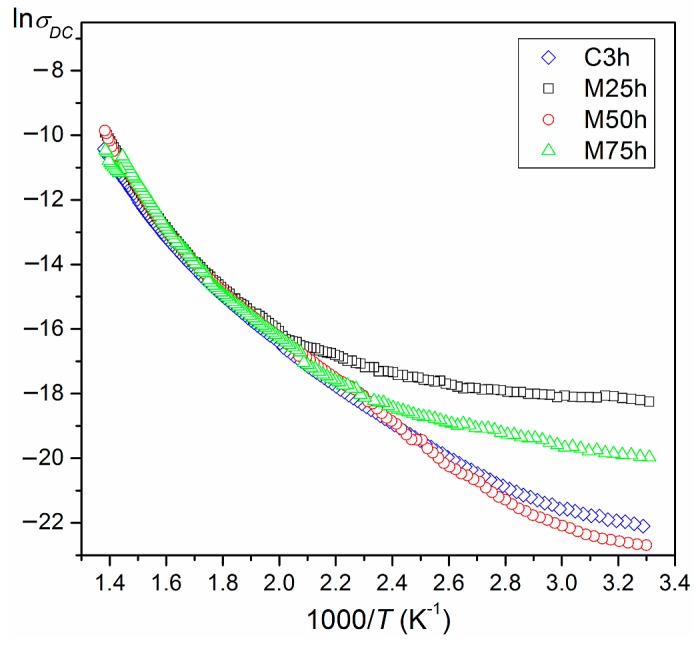
The ln*σ*(1000/*T*) relationship for the PZTMSWN ceramics.

At *T_r_*, DC resistivity of the investigated ceramic samples was of the order of magnitude from 10^7^ Ωm to 10^9^ Ωm (Table 1). Figure 7 shows the ln*σ*(1000/*T*) dependencies for the PZTMSWN ceramics. The electric conductivity increased with the increase in temperature. In the entire measurement range, the lowest electrical conductivity was found in the M50B sample, whereas the M25B sample showed the highest electrical conductivity. Based on Arrhenius’ law [31] and the slope of the linear portion of ln*σ*(1000/*T*) dependence, the activation energy in two selected areas (below and above phase transition) was calculated. The calculated values of the activation energy are listed in Table 1. 

**Figure 8:**
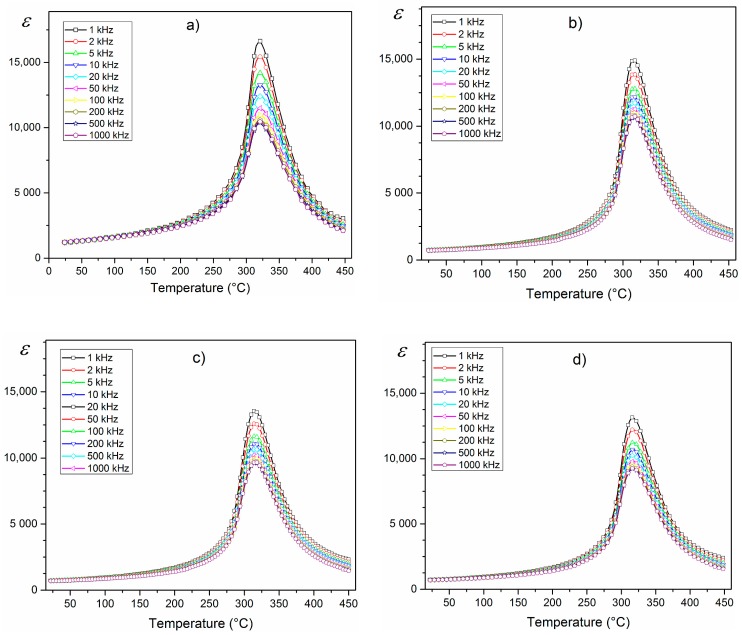
Temperature dependencies of the *ε*(*T*) for the PZTMSWN ceramics: (**a**) C3h; (**b**) M25h; (**c**) M50h; (**d**) M75h.

The temperature dependencies of *ε*(*T*) for the PZTMSWN materials are depicted in Figure 8. All ceramic samples exhibited the highest values of dielectric permitivity at *RT* and of the maximum dielectric permittivity (*ε_max_*). The investigated PZTMSWN materials did not exhibit temperature shift of the phase transition with measurement frequency. In the case of the series of samples obtained by mechanochemical synthesis (Figure 8b–d), the values of dielectric permittivity were slightly smaller than in the case of the ceramic sample obtained by classical method (Figure 8a). However, for samples obtained by mechanochemical synthesis, the phase transition occurred in a narrower temperature range. In the case of the perovskite materials, a wide temperature range of the phase transitions was related to a degree of crystalline structure ordering in the B position. When this ordering of the crystalline structure is better, the phase transitions are observed in a narrow range of temperatures. The comparision of *ε*(*T*) measured for 1 kHz for all investigated samples is shown in Figure 10a and the changes described above are clearly visible.

**Figure 9:**
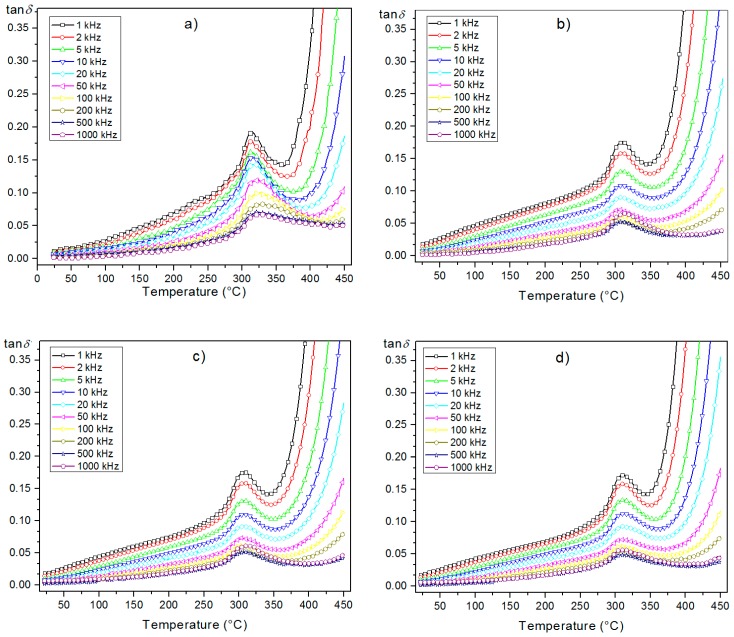
Temperature dependencies of the tan*δ* for the PZTMSWN ceramics: (**a**) C3h; (**b**) M25h; (**c**) M50h; (**d**) M75h.

**Figure 10:**
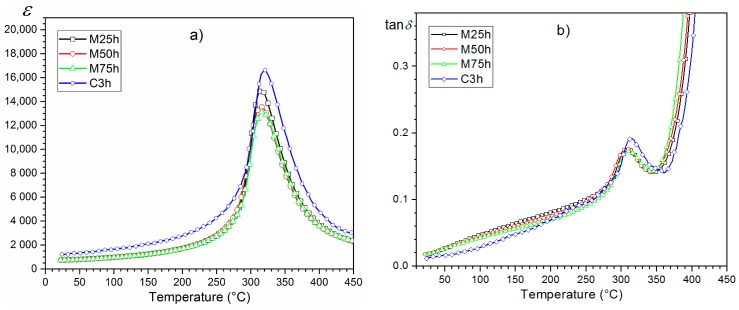
The comparision of the *ε*(*T*) (**a**) and dielectric loss (**b**) for the PZTMSWN ceramics measured at 1 kHz.

PZTMSWN samples showed low values of dielectric loss (tan*δ*) (Figure 9). The dielectric loss and the tan*δ*(*T*) exhibited similar behavior for all samples and the preparation methods did not affect dielectric properties in the entire temperature measurement area. The summary graph *ε*(*T*) for the investigated samples is depicted in Figure 10b.

**Figure 11:**
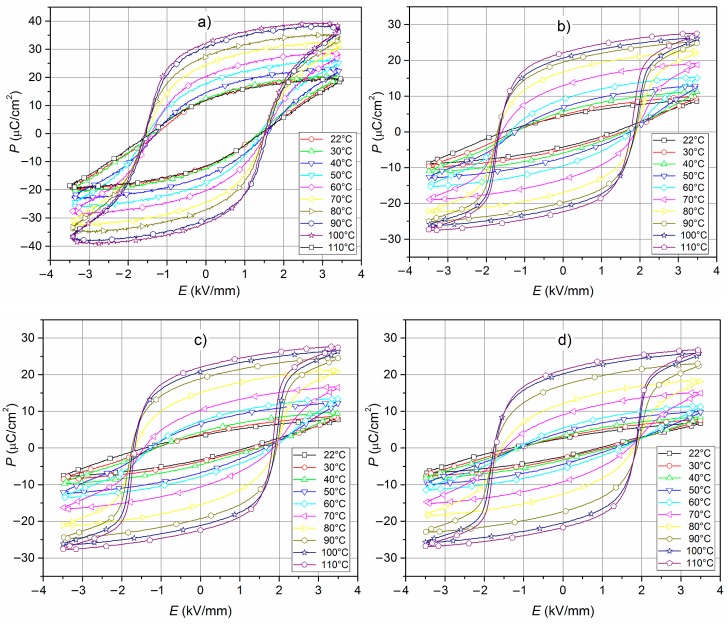
Hysteresis loops *P-E* (from 20 °C to 110 °C) of the PZTMSWN ceramics: (**a**) C3h; (**b**) M25h; (**c**) M50h; (**d**) M75h.

**Figure 12:**
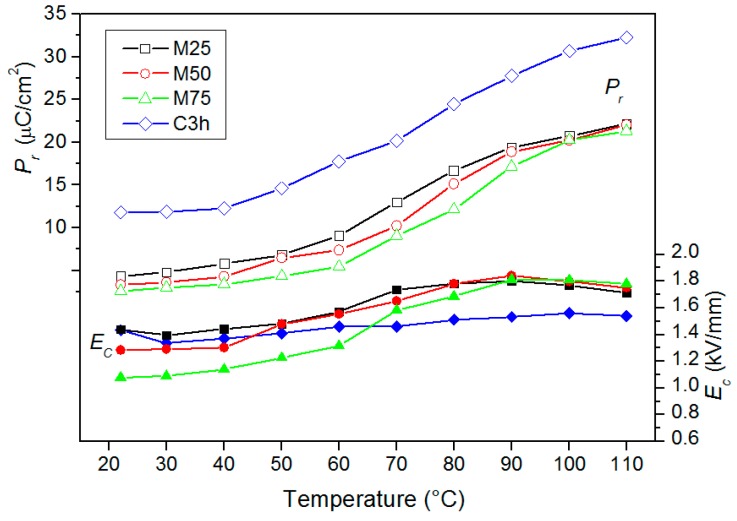
Temperature dependencies of the *P_r_* and *E_c_* of the PZTMSWN ceramic samples.

The hysteresis loops were reordered on all samples at several temperatures (Figure 11). At room temperature, the ceramic samples did not show any saturated hysteresis loops and *E_c_* coercive field from 1.08 kV/mm to 1.43 kV/mm (Table 1). In the case of the C3h sample, the value of *P_r_* remnant polarization was higher (11.80 μC/cm^2^), while the samples obtained by mechanochemical synthesis had small values of *P_r_* (from 2.60 μC/cm^2^ to 4.35 μC/cm^2^). The hysteresis loop became well saturated at the higher temperature (70–80 °C) and the remnant polarization increased. The values of the *P_r_* increased almost three times in the range from *RT* to 110 °C (Figure 12). At the same time, the *E_c_* coercive field did not change visibly (at higher temperatures there was a slight increase *E_c_*). It may be related to the used admixtures, which increased the ferroelectric properties’ stability. In the designed composition, larger amounts of the hard admixture (like Mn, Ni) were used to increase the stability of the ceramic piezoelectric parameters. In regard to the samples obtained in high-energy milling technology, the ferroelectric hysteresis loops show higher saturation observed at higher temperatures compared to the sample obtained by the conventional technology.

From the application point of view, the piezoelectric properties play the most important role. For the piezoelectric tests, all investigated ceramic samples were electrically poled by high-voltage method (in silicon oil). The values of the piezoelectric parameters were calculated using a common resonance–antiresonance method (Table 1). The PZTMSWN ceramic samples exhibited a medium level of the piezoelectric parameters, which is characteristic for hard doping of the PZT material.

## 4. Conclusions 

The ferroelectric multicomponent PZTMSWN samples with the chemical formula Pb(Zr_0.49_Ti_0.51_)_0.94_Mn_0.015_Sb_0.01_W_0.015_Ni_0.03_O_3_ were obtained. The ceramic samples were obtained by mechanochemical synthesis (after different milling periods) and the classical technology. The properties of both samples were investigated in detail and compared.

The XRD investigations confirmed the perovskite structure with the tetragonal phase at room temperature with small amounts of the pyrochlore phase. The mechanochemical synthesis of ceramic powders allows the formation of perovskite structure of the PZT-type material at low temperature (room temperature). The fine-grain powders obtained by this technology are appropriate for preparation of dense ceramic material. 

The usage of the HEBM method during the technological process of multicomponent PZT ceramics does not worsen significantly the wide spectrum of physical properties of the obtained materials; however, it significantly shortens the time of the technological process of piezoelectric ceramics (this also shortens the sintering time of the ceramic samples). The PZTMSWN ceramic samples exhibit medium values of the piezoelectric parameters, which is characteristic for hard doping of the PZT material. Lower but stable electrophysical parameters of the piezoceramic materials are extremely important for microelectronic and micromechatronic applications. The optimal dielectric parameters and good piezoelectric properties are required for ceramic elements to build actuators and piezoelectric transducers.

## Figures and Tables

**Table 1:** Physical Parameters of the PZTMSWN Ceramic Samples.

Parameter	C3h	M25h	M50h	M75h
*ρ_DC_* at *T_r_* (Ωm)	3.98 × 10^9^	8.45 × 10^7^	7.15 × 10^9^	4.70 × 10^8^
*E_Act_* in I (eV)	0.46	0.13	0.49	0.47
*E_Act_* in II (eV)	0.94	0.87	0.87	0.92
*T_C_* (°C)	320	315	316	316
*ε_r_* at *RT*	1227	745	727	726
*ε_max_* at *T_C_*	16,629	14,931	13,541	13,140
tan*δ* at *RT*	0.011	0.018	0.018	0.017
tan*δ* at *T_C_*	0.182	0.173	0.169	0.170
*P_r_* (μC/cm^2^) at *RT*	11.80	4.35	3.33	2.60
*E_c_* (kV/mm) at *RT*	1.43	1.43	1.28	1.08
*k_p_*	0.47	0.45	0.43	0.41
*d_31_* (pC/N)	52.23	52.71	54.38	44.10
*g_31_* × 10^−3^ (Vm/N)	8.00	7.37	7.68	6.48
*Q_m_*	219	235	334	497
*d_33_* (pC/N)	262	149	147	143

*RT*—room temperature.
